# A P300-Detection Method Based on Logistic Regression and a Convolutional Neural Network

**DOI:** 10.3389/fncom.2022.909553

**Published:** 2022-06-16

**Authors:** Qi Li, Yan Wu, Yu Song, Di Zhao, Meiqi Sun, Zhilin Zhang, Jinglong Wu

**Affiliations:** ^1^School of Computer Science and Technology, Changchun University of Science and Technology, Changchun, China; ^2^Zhongshan Institute of Changchun University of Science and Technology, Zhongshan, China; ^3^Research Center for Medical Artificial Intelligence, Shenzhen Institute of Advanced Technology, Chinese Academy of Sciences, Shenzhen, China

**Keywords:** brain-computer interface, convolutional neural network, electroencephalogram, event-related potential, logistic regression, P300

## Abstract

**Background:**

Electroencephalogram (EEG)-based brain-computer interface (BCI) systems are widely utilized in various fields, including health care, intelligent assistance, identity recognition, emotion recognition, and fatigue detection. P300, the main event-related potential, is the primary component detected by EEG-based BCI systems. Existing algorithms for P300 classification in EEG data usually perform well when tested in a single participant, although they exhibit significant decreases in accuracy when tested in new participants. We attempted to address this lack of generalizability associated with existing classification methods using a novel convolutional neural network (CNN) model developed using logistic regression (LR).

**Materials and Methods:**

We proposed an LR-CNN model comprising two parts: a combined LR-based memory model and a CNN-based generalization model. The LR-based memory model can learn the individual features of participants and addresses the decrease in accuracy caused by individual differences when applied to new participants. The CNN-based generalization model can learn the common features among participants, thereby reducing overall classification bias and improving overall classification accuracy.

**Results:**

We compared our method with existing, commonly used classification methods through three different sets of experiments. The experimental results indicated that our method could learn individual differences among participants. Compared with other commonly used classification methods, our method yielded a marked improvement (>90%) in classification among new participants.

**Conclusion:**

The accuracy of the proposed model in the face of new participants is better than that of existing, commonly used classification methods. Such improvements in cross-subject test accuracy will aid in the development of BCI systems.

## Introduction

Electroencephalogram (EEG)-based brain-computer interface (BCI) systems use brain signals to transmit information. The primary feature of these systems is that self-elicited EEG signals can assist patients with loss of motor function in the limbs and those with language impairments in communicating with others by enabling them to control external devices. The BCI system has gradually moved from the laboratory to the market, which requires a stronger adaptability of the BCI system (Hochberg et al., 2006; Hoffmann et al., 2008; Xu et al., 2021).

The study of EEG classification problems is important for the application and development of the BCI system (Hu et al., 2019; Hu and Zhang, 2020; Li, 2020; Li et al., 2020). In EEG, event-related potentials (ERPs) are specific voltage signals generated in the brain in response to a task (e.g., gazing at numbers, letters, or pictures). Currently, diseases such as Alzheimer’s disease can be studied as an aid to identify early cognitive impairment by studying changes in patients’ ERPs, and the study of ERPs is important for medical rehabilitation, medical diagnosis, and improving EEG-based communication systems (Luck, 2005; Wang et al., 2017; Yan et al., 2018; Liu et al., 2022). Lu et al. (2020) constructed time-varying networks for ERPs in AV and V spelling paradigms based on adaptive directed transfer function to investigate the dynamic processes underpinning the processing of stimuli in the two spelling paradigms. The P300 potential, which is usually detected about 300 ms after the appearance of the target stimulus (Farwell and Donchin, 1988; Rakotomamonjy and Guigue, 2008), is generally used as the primary reference potential. Lu et al. (2019) designed a novel audiovisual P300-speller paradigm to improve the performance of vision-based P300 spelling system. P300 data are usually represented as a matrix with two dimensions (channel and time), and their values represent the true EEG amplitude obtained from the individual during task performance (Rakotomamonjy and Guigue, 2008). Li et al. (2019) attempt to improve the performance of P300 by increasing the user’s mental work. The traditional EEG classification process includes preprocessing, feature extraction, and classification (Rashid et al., 2019). Liu et al. (2021) proposed a sparse representation of the P300 spelling paradigm to solve the problem of brain signal classification with high-dimensional data and low signal-to-noise ratio. Preprocessing usually includes filtering, baseline correction, and artifact removal (Kundu and Ari, 2020). Feature extraction allows one to obtain the most discriminative features from real EEG data (Dodia et al., 2019). Traditional feature extraction generally extracts features from the time domain (e.g., variance, mean, and kurtosis) (Martin-Smith et al., 2017), frequency domain (e.g., fast Fourier transformation) (Park and Chung, 2018), and time-frequency domain (e.g., discrete wavelet transformation) (Amini et al., 2013).

Machine learning and deep learning have been widely used in image recognition (Liu et al., 2019), medical diagnosis (Zhao et al., 2022), intelligent assistance and other fields. In recent years, these methods have also become a hot topic in P300 signal detection. The P300 EEG signal is a complex combination of superimposed multi-band waveforms, and a number of classification methods have been used to decode the P300 signal. For example, principal component analysis (PCA) and local Fisher discriminant analysis (LFDA) are commonly used to reduce the dimensionality of features (Bernat et al., 2007), while linear discriminant analysis (LDA) (Dodia et al., 2019), support vector machine (SVM) (Li et al., 2014), decision tree (DT) (Guan et al., 2019), random forest (RF) (Akram et al., 2015; Masud et al., 2018), ADB (Hongzhi et al., 2012; Yildirim and Halici, 2014), and k-nearest neighbor (k-NN) methods (Guney et al., 2021) are commonly used for P300 classification.

Since traditional preprocessing and feature extraction methods are highly complex and time-consuming and can lead to a loss of important information after extraction, automated feature extraction algorithms are considered important (Shan et al., 2018). Deep learning represents a better solution to this problem (Oralhan, 2020), as it provides an excellent algorithm for automatically extracting discriminative features. Deep learning is currently adopted in many studies, as it can learn features from the original data well (Kshirsagar G. and Londhe N., 2019; Kshirsagar G. B. and Londhe N. D., 2019; Borra et al., 2021). At present, the main deep learning algorithms used for EEG data processing include convolutional neural networks (CNNs), recurrent neural networks (RNNs), deep belief networks (DBN), autoencoders (AEs), and other models (Maddula et al., 2017; Lu et al., 2018). Cecotti et al. introduced a CNN for detecting the P300 ERP in BCIs (Cecotti and Gräser, 2011). They proposed seven CNN-based classifiers and evaluated their performance, with excellent results. Feng et al. used a CNN classification algorithm based on PCA to classify P300 data (Li et al., 2020). Ditthapron et al. used multi-task AE-based feature extraction for EEG classification (Ditthapron et al., 2019). Vařeka et al. used stacked AEs for P300 classification (Vařeka and Mautner, 2017). Since the RNN model has achieved good results in sequential information recognition tasks (such as speech recognition) (Lipton et al., 2015), long short-term memory (LSTM) networks have also been applied to EEG recognition. Joshi et al. proposed a neural network model based on convolutional long short-term memory (ConvLSTM), in which a CNN and LSTM were used to capture spatial and temporal information, respectively. This effective use of temporal as well as spatial features yielded better performance than a single system (Joshi et al., 2018). Kundu et al. proposed a PCA-based ensemble of a weighted SVM (PCA-EWSVM) classifier. In this weighting method, different weights were assigned to each classifier, with the largest weight assigned to the best classifier, causing it to have the greatest impact on the final output of the classifier (Kundu and Ari, 2017). Kshirsagar et al. constructed a weighted ensemble using a deep CNN, which effectively reduced the classification error rate for single models, and adopted a new channel dropout-based character-detection approach, which further reduced the false detection rate arising from a single trial (Krusienski et al., 2008). Feng et al. proposed an automatic P300 EEG signal channel selection algorithm based on population sparsity Bayesian logistic regression (Feng and Zhang, 2019). Abibullaev et al. proposed multiple network structures for P300 signal analysis to select a more accurate classifier to decode the signal, however, the complexity of the network structure leads to higher complexity of the algorithm and more parameters, which makes it not suitable for online applications (Abibullaev and Zollanvari, 2021). Xu et al. proposed an online pre-alignment strategy for motion imagery signals to address the generalization ability of the model across datasets and obtained good results, and in 2021, this team made further improvements by combining adaptive batch normalization (AdaBN) and alignment strategies to reduce interval covariate shifts between datasets, but this approach was used more for processing datasets before validation and its insensitive to different individual features when used for P300 signal processing (Xu et al., 2020; Xu et al., 2021).

Stochastic gradient descent (SGD) is commonly utilized to optimize deep learning algorithms (Bottou, 2010). In 2009, John Duchi proposed the FOBOS (Forward-Backward Splitting) optimization algorithm (Duchi and Singer, 2009a,b), which divides the regularized gradient descent problem into an empirical loss gradient descent iteration and an optimization problem. However, FOBOS only uses the gradient of the previous iteration, without accumulation, and does not achieve effective sparsity. In 2010, Lin et al. proposed the regularized dual averaging (RDA) optimization algorithm (Xiao, 2010), which relies on gradient accumulation. In this algorithm, when the average value of accumulated gradients on a latitude is less than a threshold, the weight of that latitude will be set to zero, which ensures that the weights are fully trained. In 2011, McMahan et al. proposed the Follow the Regulation Leader (FTRL) algorithm (McMahan, 2011), which has worked well in many recommender systems (McMahan et al., 2013; Kim, 2017). FTRL is a general optimization algorithm for online deep learning prediction, which inherits the idea of SGD. Based on a truncated gradient, it absorbs the advantages of the FOBOS and RDA optimization algorithms, focusing on the sparsity problem while improving accuracy, which has great potential for application to online prediction systems.

Previous studies have shown that the amplitude and latency of the P300 component vary across individuals according to sex and age (Carlson and Iacono, 2006; Brumback et al., 2012; Dinteren et al., 2014). Therefore, an EEG-processing algorithm should have certain memorization and generalization capabilities for individuals of different ages and sexes to allow it to discover the common features among participants and effectively aid in subsequent classification. When we tested the existing commonly used algorithms, we found that they performed well on a single dataset (>95% accuracy). However, when we tested them on other available datasets and private datasets, the accuracy dropped significantly to around 80%.

To address this sharp decrease in accuracy when existing algorithms are applied to new datasets, we proposed an LR-CNN model that combines logistic regression (LR) and a CNN. In this model, optimization is achieved via the FTRL algorithm, which addresses the sparsity problem, and the network is applied to P300 detection, which also addresses sparse data features and improves the robustness of the algorithm across participants. We conducted experiments using a private dataset and compared our results with those of previous studies to determine whether our proposed method can achieve better results in the detection of the P300 component between different individuals.

## Materials and Methods

### Participants

This study was approved by the Ethics Committee of Changchun University of Science and Technology. All participants provided written informed consent after receiving a detailed explanation of the experimental procedure. Eight healthy, right-handed individuals with no mental disorders, normal or corrected-to-normal vision, and a stereopsis acuity better than 60″ participated in the study.

### Paradigm Flow

Preparation for the EEG experiment included placing electrode caps on each participant, applying electrode paste to reduce the resistance to a reasonable range, and informing the participants of the experimental procedure and tasks.

**FIGURE 1:**
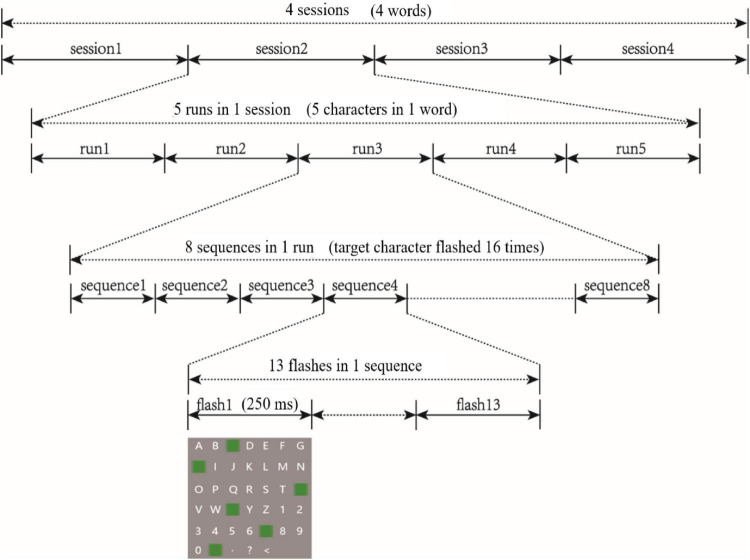
P300 speller paradigms.

We prepared two-dimensional and three-dimensional P300 speller paradigms, which were presented to participants using an NVIDIA stereo display with a resolution of 1,920 × 1,080 and a refresh rate of 120 Hz. Following EEG preparations, participants sat directly in front of the stereo display with their eyes 90 cm from the screen. Each task involved four sessions in which a word containing five characters was presented. Each session contained five runs, and each run output one character. The two tasks included a total of eight sessions. To avoid learning effects, the eight sessions were presented in pseudo-random order for each participant. Because the task paradigm was a 6 × 7 matrix of characters, the target character flashed twice every 13 flashes (once in row, once in column), which was called a sequence. Each run included eight sequences (i.e., a target character was output by flashing 104 times, as shown in Figure 1). During the tasks, participants were required to stare at the target character and silently count the number of times it flashed. They were permitted a 5-min rest period after each session.

### Dataset Processing

The EEG data used in this study were collected during the experiment using 64 active electrodes. Considering the amount of data requiring processing, we selected 30 standard electrodes according to the International 10-20 electrode system as nodes to build the brain networks, including FP1, FPz, FP2, F7, F3, Fz, F4, F8, FC3, FCz, FC4, T7, C3, Cz, C4, T8, CP3, CPz, CP4, P7, P3, Pz, P4, P8, PO5, POz, PO6, O1, Oz, and O2. The raw EEG data of each participant were preprocessed for the time-varying brain network analysis.

The raw EEG data were bandpass filtered between 0.1 and 30 Hz with a third-order Butterworth filter. The EEG was segmented according to the coding value of each Flash marker, from 100 ms before to 800 ms after the presentation of the stimuli, with a duration of 900 ms, for analysis. The data between −100 and 0 ms was used for baseline correction. According to the characteristics of the brainwaves, a threshold of −100 to 100 μV was used for artifact removal.

In the prediction phase, the data for the previous 1 s were collected every 0.5 s and input into the model.

### Model

The model proposed in this study comprised two parts: a memorizing model and a generalizing model. The memorizing model was defined as an information retrieval model, expressed as follows:


(1) $$f\left(x\right)=\mathrm{sigmoid}\left(w^{\text{T}}x+b\right),x=\left(x_{1},x_{2},\cdots ,x_{d_{1}+d_{2}+d_{\phi }}\right)$$

where *w*^T^ = (*w*_1_, *w*_2_ … *w*_*d*_1_+*d*_2_+*d*_φ__) represents the regression coefficient, *b* represents the bias, *x* = (*x*_1_, *x*_2_, ⋯ , *x*_*d*_1_+*d*_2_+*d*_φ__) represents the matrix of independent variables, *d*_*1*_ represents the dense EEG data, *d*_*2*_ represents the sparse (one-hot encoding) feature data of the participant, and *d*_φ_ represents the data after cross-product transformation, which was defined as follows:


(2) $$\psi \left(x\right)=\prod_{i=1}^{d}x_{i=1}^{c_{\mathrm{ki}}},c_{\mathrm{ki}}\in \left\{0,1\right\}$$

In Eq. (2), *c*_*ki*_ is a Boolean value that serves to control the multiplication of specific features.

The prediction function is a Sigmoid function as shown in Eq. (3).


(3) $$f\left(z\right)=\frac{1}{1+e^{-z}}Z\in \left(-\infty ,+\infty \right)\;$$

This function controls the prediction value between (0,1) and makes the classification for judging the P300 signal.

**FIGURE 2:**
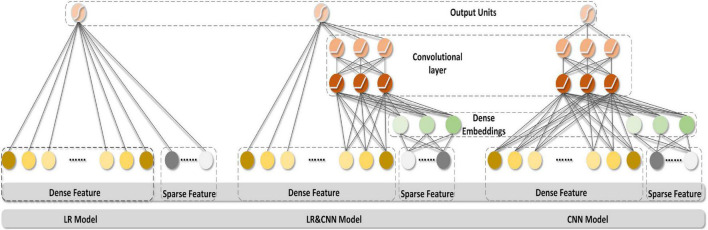
Structure diagrams of the models.

The generalizing model was a CNN, as shown in Figure 2. Tables 1, 2 show the network parameters. The original W&D model used the multilayer perceptron (MLP) network. Since the EEG signal was dense with features in each frequency band, and the CNN can efficiently analyze the signal data with a fixed length, a multilayer one-dimensional CNN was selected as the generalizing model. The model structure with the best result, which had a filter of 1 × 3 and three layers, was obtained through exhaustive selection.

**TABLE 1:** Parameter table for CNN section.

Layer	Filters	Size	Params	Activation	Feature map size
Input(EEG)		(batch_size, 30,90)			
Input(Spare Feature)		(batch_size,1,T)			
Embedding[Input(Spare Feature)]		output(1,90)	90*T		
Concatenate		(batch_size,31,90)			
Conv2D	16	(batch_size,1,7)	16*1*7 + 16	Leaky Relu	31*42*16(step = 2)
Conv2D	32	(1,7)	32*1*7*16 + 32	Leaky Relu	31*18*32(step = 2)
Conv2D	16	(1,7)	16*7*32 + 16	Leaky Relu	31*13*16(step = 2)
Flatten		(6448,1)			
Dense	*N*			Sigmoid	

*L, number of cross-feature; N, number of classes.*

**TABLE 2:** Parameter table for LR section.

Layer	Size	Params	Activation
Input(EEG)	(30,90)		
Flatten	(2700,1)		
Linear	(1,2700) (1)	2,701	
Dense	*N*		Sigmoid

#### Optimization Algorithm

The FTRL algorithm was used to optimize our mixed model. The FOBOS algorithm introduces L1 regularization based on gradient descent and sets a threshold for parameter truncation; however, the threshold slowly becomes smaller as the number of iterations increases and needs to be improved in sparsity.

The FTRL algorithm combines the advantages of the SGD and FOBOS algorithms, inheriting the accuracy and good sparsity of weights of the updated SGD algorithm from the FOBOS algorithm (Duchi and Singer, 2009b). This led to good real-time performance of our EEG classification model. The weight-updating formula was as follows:


$$W_{t+1}=\underset{W}{argmin}$$

(4) $$\left\{G_{1:t}\text{⋅W}+\frac{1}{2}\sum_{\text{s}=1}^{\text{t}}\sigma_{\text{s}}|{\left|W-W_{\text{s}}\right|{|}_{2}^{2}+\lambda_{1}|\left|W\right||}_{1}+\frac{1}{2}\lambda_{2}{||W||}_{2}^{2}\right\}$$

where *G*_*1:t*_ represents the sum of the gradients accumulated up to time *t*, σ_*s*_ represents the learning rate (which contained two parameters to be input, known as Per-Coordinate Learning Rates), and λ_1_||*W*||_1_ and $\lambda_{2}||W||{}_{2}^{2}$ represent the regulars of L1 and L2, respectively. The purpose of L1 regularization was to increase the sparsity of network weights, allowing for real-time recognition of the EEG data. The purpose of L2 regularization was to increase the smoothness of the results in the optimization process. The FTRL algorithm updated each Dim of W separately (i.e., using different learning rates, which accounted for the uneven feature distribution of the data). If there were few features in a certain dimension of a certain data (i.e., the feature value was 0), every such sample would become very important, and the corresponding learning rate of the feature value could be kept very large. The specific weight update algorithm is shown in Algorithm 1.

**Algorithm 1:** FTRL with L1 and L2 regularization.

1. *input* α, β, λ_1_, λ_2_
2. *initialize W* ∈ ℝ^*N*^, *Z* = 0 ∈ ℝ^*N*^, *Q* = 0 ∈ ℝ^*N*^
3. *for t* = 1, 2, 3 … *do*
4. *G* = ∇_*w*_*L*(*W*, *X*^(*t*)^, *y*^(*t*)^)
5. *for i* = 1, 2, 3 …, *N do*
6. $\sigma_{i}=\frac{1}{\alpha }\sqrt{q_{i}+g_{i}^{2}}-\sqrt{q_{i}}\&q_{i}=q_{i}+g_{i}^{2}$
7. *z*_*i*_ = *z*_*i*_ + *g*_*i*_ − σ_*i*_*w*_*i*_
8. $w_{i}=\{\begin{array}{c} 0\;if\left|z_{i}^{\left(t\right)}\right|<\lambda_{1}\\ -{\left(\lambda_{2}+\frac{\beta +\sqrt{q_{i}}}{\alpha }\right)}^{-1}\left(z_{i}-\lambda_{1}sgn\left(z_{i}\right)\right)\;otherwise\; \end{array}$
9. *end*
10. *end*
11. *return W*

#### Training Phase

In the training phase, FTRL was used as the optimizer, while logistic loss was used to represent loss. The loss function of logistic regression was formulated as follows:


(5) $$\begin{array}{c} J\left(w\right)=-\frac{1}{m}\sum_{i=1}^{m}y^{\left(i\right)}\log\left(\sigma \left({\mathrm{wx}}^{\left(i\right)}+b\right)\right)+\\ \left(1-y^{\left(i\right)}\right)\log\left(1-\sigma \left({\mathrm{wx}}^{\left(i\right)}+b\right)\right)\\ ,x=\left(x_{1},x_{2},\cdots ,x_{d_{1}+d_{2}+d_{\varphi }}\right) \end{array}$$

Suppose there are *m* samples. In this case, *x*^(*i*)^ denotes any certain sample, the dimension of the sample is *d*_1_ + *d*_2_ + *d*_φ_, σ(⋅) denotes the sigmoid function, and w is the parameter vector required to solve such that (*w^T^x*_*i*_ + *b*) yields the probability prediction value in the sigmoid function. *y*^(*i*)^ denotes the prediction value of the *i*th sample. When *y* = 1, the latter equation is 0; when *y* = 0, the previous equation is 0. The loss functions for predicting positive and negative values are combined by *y*^(*i*)^, and the average of *m* samples is taken to finally obtain the loss function of logistic regression.

#### Prediction Phase

Since the prediction phase included data for a single participant, the embedding layer only needed to be calculated once (because the sparse TENSOR recorded the participant FEATURE). Therefore, the model we proposed would exhibit good real-time performance and predictive robustness.

##### Prediction Formula


(6) $$P\left(Y=1|x\right)=\sigma \left(w_{\mathrm{wide}}^{T}\left[x,\phi \left(x\right)\right]+w_{\mathrm{Conv}}a^{l_{f}}+b\right)$$

where *Y* is the binary class label, σ(⋅) is the sigmoid function, φ(*x*) are the cross-product transformations of the original features *x*, and *b* is the bias term. Further, in this equation, $w_{wide}^{T}$ is the vector of all wide model weights, and *w*_*Conv*_ are the weights applied on the final activations *a^l_f_^*.

## Results

We conducted three experiments to verify the effectiveness of the proposed model. We performed 5-fold cross-validation and took the average as the result. In the first set of experiments, data from eight participants were collected every other day to train and test the model. The data of each participant were divided into a training set (80%) and a test set (20%). Each classifier was trained using the training set, following which the test was conducted using the test set. The test results are shown in Table 3. Among them, the test accuracy of the DT for each participant was generally low, with an accuracy rate of only around 80%. The test accuracy rate of LR was only 82.5% in the first participant, but the test accuracy rates in the remaining seven participants were all above 90%. The test accuracy rates of RF, Adboost (ADB), MLP, SVM, KNN algorithm, and LDA in each participant all exceeded 90%. The CNN and LSTM approaches performed very well for a single participant, with accuracies of over 95%. Our proposed model achieved an accuracy rate greater than 90% on the test set in each participant, reaching up to 95.6%.

**TABLE 3:** Test accuracy when training individual participants separately.

	DT	RF	ADB	LR	MLP	SVM	KNN	LDA	LR-CNN	CNN	LSTM
Participant 1	0.819	0.931	0.932	0.825	0.936	0.931	0.906	0.931	0.951	0.989	0.959
Participant 2	0.826	0.933	0.933	0.933	0.934	0.936	0.919	0.933	0.942	0.978	0.968
Participant 3	0.838	0.932	0.932	0.932	0.933	0.934	0.931	0.932	0.929	0.992	0.962
Participant 4	0.828	0.935	0.935	0.935	0.935	0.935	0.928	0.935	0.946	0.992	0.972
Participant 5	0.816	0.933	0.933	0.933	0.933	0.936	0.915	0.933	0.919	0.984	0.954
Participant 6	0.767	0.934	0.934	0.934	0.934	0.935	0.917	0.933	0.956	0.977	0.961
Participant 7	0.820	0.935	0.936	0.935	0.935	0.936	0.915	0.835	0.927	0.969	0.959
Participant 8	0.788	0.932	0.932	0.932	0.932	0.932	0.920	0.932	0.933	0.996	0.967

*DT, decision tree; RF, random forest; ADB, adboost; LR, logistic regression; MLP, multilayer perceptron; SVM, support vector machine; KNN, k-nearest neighbor; LDA, linear discriminant analysis; LR-CNN, logistic regression and convolutional neural network; CNN, convolutional neural network; LSTM, long short-term memory.*

In the second set of experiments, data from seven participants were collected every other day to train the model and tested on the remaining participant. The test results are shown in Table 4. Since the test participant was never seen in the training set, the test accuracy rate of the traditional classification methods dropped significantly. Among them, the accuracy rate of DT decreased from around 82% to around 73%, and the accuracy rates of LR, RF, ADB, MLP, SVM, KNN, and LDA decreased from over 90 to 80-85%. The accuracy of the CNN and LSTM approaches also decreased from a very high 95%+ to 85–90%, with more dramatic decreases observed across participants. Because the classifier had not learned some individual features, the accuracy rate decreased significantly when used in participants it had not encountered previously. However, since the wide part and embedding layer of the proposed LR-CNN model had learnt some individual features, the test accuracy did not decrease significantly when the model was applied to new individuals. When data for participant 7 were tested, the accuracy of the model decreased markedly when compared with that for other participants, indicating that the individual features of participant 7 did not appear in the dataset for the other seven participants.

**TABLE 4:** Test results for the remaining participant after training using data for the other seven participants.

	DT	RF	ADB	LR	MLP	SVM	KNN	LDA	LR-CNN	CNN	LSTM
Participant 1	0.759	0.825	0.828	0.793	0.856	0.829	0.806	0.841	0.923	0.898	0.886
Participant 2	0.737	0.836	0.828	0.813	0.863	0.837	0.819	0.824	0.932	0.883	0.877
Participant 3	0.742	0.831	0.829	0.798	0.856	0.835	0.816	0.823	0.919	0.869	0.855
Participant 4	0.716	0.822	0.826	0.816	0.853	0.840	0.814	0.854	0.932	0.889	0.883
Participant 5	0.732	0.836	0.833	0.822	0.833	0.833	0.811	0.821	0.917	0.796	0.811
Participant 6	0.721	0.820	0.827	0.821	0.843	0.836	0.810	0.826	0.926	0.913	0.878
Participant 7	0.727	0.843	0.826	0.816	0.852	0.842	0.812	0.822	0.862	0.877	0.858
Participant 8	0.712	0.832	0.829	0.794	0.829	0.828	0.821	0.829	0.921	0.881	0.862

*DT, decision tree; RF, random forest; ADB, adboost; LR, logistic regression; MLP, multilayer perceptron; SVM, support vector machine; KNN, k-nearest neighbor; LDA, linear discriminant analysis; LR-CNN, logistic regression and convolutional neural network; CNN, convolutional neural network; LSTM, long short-term memory.*

In the third set of experiments, data from four of the eight participants were randomly selected for training, and data from these four individuals were collected every other day to train the model, which was tested on data for the remaining four participants. To avoid randomness of the results, we carried out the process of randomly selecting four participants for training and the remaining four participants for testing a total of four times, as shown in Tables 5–8. The overall accuracy rates of DT, LR, RF, ADB, MLP, SVM, KNN, LDA, and LSTM further decreased slightly when compared with those observed in the second set of experiments. This was due to the reduction of training data (from seven participants to four participants, whose data were not used during training). However, test accuracy for the four participants did not decrease when our proposed model was applied, and all accuracy rates exceeded 90% (Table 5).

**TABLE 5:** Test results for the remaining four participants after training using data from four participants (1).

	DT	RF	ADB	LR	MLP	SVM	KNN	LDA	LR-CNN	CNN	LSTM
Participant 1	0.656	0.811	0.808	0.763	0.816	0.817	0.785	0.824	0.913	0.839	0.822
Participant 5	0.707	0.806	0.798	0.773	0.823	0.815	0.801	0.815	0.926	0.828	0.802
Participant 6	0.773	0.831	0.793	0.787	0.808	0.820	0.800	0.818	0.915	0.779	0.798
Participant 8	0.703	0.802	0.806	0.801	0.824	0.833	0.796	0.820	0.909	0.818	0.802

*DT, decision tree; RF, random forest; ADB, adboost; LR, logistic regression; MLP, multilayer perceptron; SVM, support vector machine; KNN, k-nearest neighbor; LDA, linear discriminant analysis; LR-CNN, logistic regression and convolutional neural network; CNN, convolutional neural network; LSTM, long short-term memory.*

**TABLE 6:** Test results for the remaining four participants after training using data from four participants (2).

	DT	RF	ADB	LR	MLP	SVM	KNN	LDA	LR-CNN	CNN	LSTM
Participant 3	0.743	0.811	0.810	0.746	0.814	0.809	0.771	0.819	0.913	0.811	0.798
Participant 4	0.729	0.815	0.805	0.791	0.817	0.822	0.809	0.822	0.873	0.832	0.822
Participant 7	0.716	0.824	0.803	0.814	0.816	0.821	0.818	0.827	0.857	0.841	0.836
Participant 8	0.704	0.811	0.806	0.801	0.816	0.836	0.766	0.808	0.861	0.856	0.843

*DT, decision tree; RF, random forest; ADB, adboost; LR, logistic regression; MLP, multilayer perceptron; SVM, support vector machine; KNN, k-nearest neighbor; LDA, linear discriminant analysis; LR-CNN, logistic regression and convolutional neural network; CNN, convolutional neural network; LSTM, long short-term memory.*

**TABLE 7:** Test results for the remaining four participants after training using data from four participants (3).

	DT	RF	ADB	LR	MLP	SVM	KNN	LDA	LR-CNN	CNN	LSTM
Participant 2	0.750	0.801	0.805	0.820	0.808	0.824	0.862	0.821	0.933	0.812	0.801
Participant 3	0.707	0.823	0.804	0.766	0.801	0.821	0.801	0.810	0.912	0.833	0.826
Participant 4	0.724	0.804	0.833	0.793	0.823	0.809	0.796	0.803	0.929	0.815	0.833
Participant 7	0.717	0.829	0.806	0.817	0.811	0.815	0.807	0.834	0.882	0.822	0.812

*DT, decision tree; RF, random forest; ADB, adboost; LR, logistic regression; MLP, multilayer perceptron; SVM, support vector machine; KNN, k-nearest neighbor; LDA, linear discriminant analysis; LR-CNN, logistic regression and convolutional neural network; CNN, convolutional neural network; LSTM, long short-term memory.*

**TABLE 8:** Test results for the remaining four participants after training using data from four participants (4).

	DT	RF	ADB	LR	MLP	SVM	KNN	LDA	LR-CNN	CNN	LSTM
Participant 1	0.701	0.801	0.818	0.793	0.816	0.827	0.810	0.821	0.923	0.846	0.839
Participant 4	0.718	0.816	0.801	0.778	0.807	0.802	0.801	0.804	0.882	0.883	0.806
Participant 6	0.744	0.822	0.786	0.782	0.818	0.809	0.797	0.818	0.919	0.816	0.816
Participant 8	0.702	0.835	0.769	0.762	0.803	0.789	0.783	0.801	0.861	0.855	0.841

*DT, decision tree; RF, random forest; ADB, adboost; LR, logistic regression; MLP, multilayer perceptron; SVM, support vector machine; KNN, k-nearest neighbor; LDA, linear discriminant analysis; LR-CNN, logistic regression and convolutional neural network; CNN, convolutional neural network; LSTM, long short-term memory.*

**FIGURE 3:**
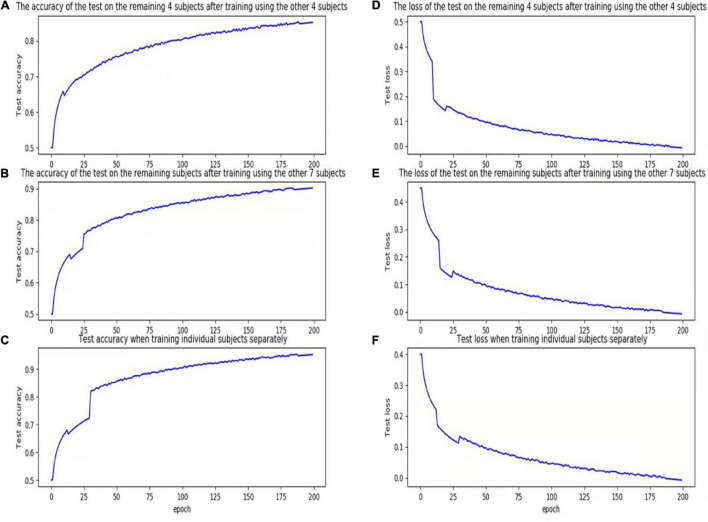
Accuracy and loss figures for training. **(A)** The accuracy of the test on the remaining 4 subjects after training using the other 4 subjects. **(B)** The accuracy of the test on the remaining subjects after training using the other 7 subjects. **(C)** Test accuracy when training individual subjects separately. **(D)** The loss of the test on the remaining 4 subjects after training using the other 4 subjects. **(E)** The loss of the test on the remaining subjects after training using the other 7 subjects. **(F)** Test loss when training individual subjects separately.

We plotted the training loss and test accuracy, as shown in Figure 3. Table 6 shows that the test accuracy of the proposed model was significantly reduced in test participants 4, 7, and 8. In the second set of experiments, the individual features of participant 7 did not appear in the other seven participants. For the analyses summarized in Tables 5, 7, the test accuracy rates did not significantly decrease in participants 4 and 8, although relatively significant decreases were observed in the analyses summarized in Tables 6, 8. This was because participants 4 and 8 shared relatively similar features that were not observed in other participants. Therefore, if data for participants 4 and 8 were not included in the training phase but were only part of the testing phase, the accuracy would decrease significantly. However, when compared with that for the traditional learning methods, the test accuracy rate was still relatively high for our proposed model, indicating that our model also learned some individual features.

The above experiments demonstrated that the proposed model can better learn individual participant features when compared with traditional classification methods, with increasing generalization capability as the size of the training set (i.e., number of participants) increases.

## Discussion

In this study, the proposed LR-CNN model exhibited better accuracy in the test phase than existing methods that are commonly used for P300 classification.

**FIGURE 4:**
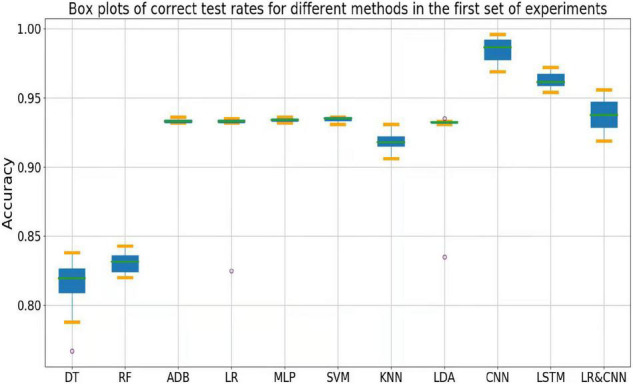
Box plots of test accuracy for different methods in the first set of experiments.

**FIGURE 5:**
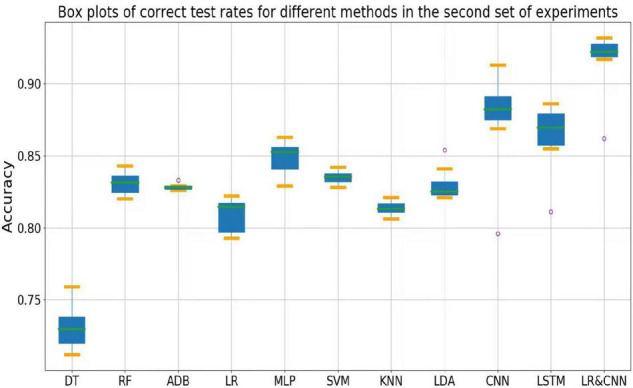
Box plots of test accuracy for different methods in the second set of experiments.

**FIGURE 6:**
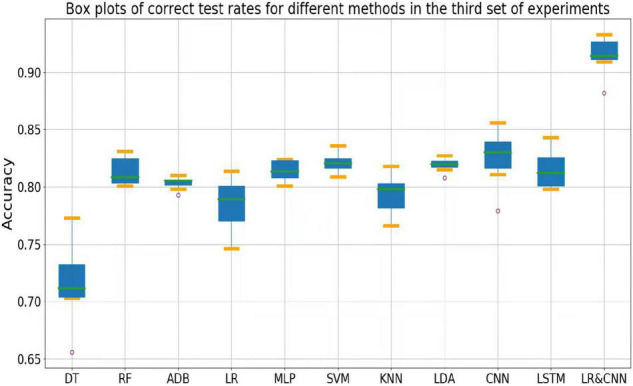
Box plots of test accuracy for different methods in the third set of experiments.

The accuracy rates of each method in the three sets of experiments are shown in the box plots below. Figure 4 shows a box plot of the accuracy rate for each method in the first set of experiments, Figure 5 represents that in the second set of experiments, and Figure 6 represents those in the third set of experiments. The accuracy rates of DT in the three sets of experiments were generally low, those for strong classifiers such as RF, ADB, MLP, and SVM were relatively high in the first set of experiments, with very little fluctuation. This indicated that these classifiers had learnt the common features of the eight participants, leading to a stable and relatively high accuracy rate when testing these participants. In particular, the CNN and LSTM approaches, which learn features better than other classifiers, perform very well when applied to a single participant. However, the overall accuracy rates of these methods decreased significantly in the second and third sets of experiments, with large fluctuations. Due to the differences across participants, the common features learned by these strong classifiers and CNN/LSTM had different applicability to each new participant, leading to large fluctuations in accuracy rates across testers and an overall decrease in test accuracy. These results suggest that these strong classifiers and CNN/LSTM had not learned the individual features of the participant.

Outliers were observed when LR and LDA were applied in the first set of experiments, but the overall classification accuracy rates were relatively high with very little fluctuation when the outliers were excluded. This indicated that these two classifiers may have strengthened some common feature types and that some individuals were not sensitive to such features, leading to the presence of outliers in which the accuracy rate was much lower than the average. In the second and third sets of experiments, as for the above-mentioned strong classifiers, the overall accuracy rates of these two classifiers decreased significantly and exhibited large fluctuations across participants. This can also be explained by the failure of the classifiers to learn the individual features of participants.

As shown in Figure 4, the classification accuracy of the CNN and LSTM approaches exceeded that of the traditional approach in a single participant, slightly outperforming our approach. However, as shown in Figures 5, 6, the classification accuracies of these two methods continued to decrease during cross-participant testing. Moreover, as shown in Figure 6, the classification accuracy of CNN and LSTM further decreased as the number of participants decreased relative to that shown in Figure 5. Thus, the CNN and LSTM approaches are limited by the numbers of cross-participant tests and participants in general.

Our LR-CNN classifier maintained a high level of overall accuracy in the three sets of experiments, with no significant decrease for new participants. In the first set of experiments, when compared with that for other methods, the accuracy of our model fluctuated more across participants with a relatively high average accuracy rate, which suggested that our model learned both common and individual features. Due to the different degrees of learning for individual features, the accuracy rate fluctuated greatly across participants when compared with that observed using other models. However, in the second and third sets of experiments, the overall degree of fluctuation and accuracy rate did not change significantly when compared with those for other models, further suggesting that our model learned both common and individual features. The outliers in the second and third sets of experiments arose from individual features of these participants that differed from those of participants in the training set. Thus, the training samples did not have relevant individual features for our model to learn. This also highlights the adaptability of our model: As the number of individual participants increases, the model can learn more individual features, thus continuously increasing its generalizability.

### Limitations

First, our test dataset was small, meaning that our method has a limited ability to learn individual features. A small number of participants in the training set would result in some individual features not being included, reducing our classifier’s accuracy rate when testing these participants. Second, although our model is capable of learning more features as the number of participants increases, further studies with larger datasets are required to verify its value.

## Conclusion

Our proposed LR-CNN model exhibited better test accuracy than existing methods that are commonly used for classification. Such improvements in the accuracy of extracting features from EEG data will be useful for the development of BCI systems.

## Data Availability Statement

The raw data supporting the conclusions of this article will be made available by the authors, without undue reservation.

## Ethics Statement

Written informed consent was obtained from the individual(s) for the publication of any potentially identifiable images or data included in this article.

## Author Contributions

YW contributed to the conception of the study, assisted with data analysis, and participated in constructive discussions. YS and DZ wrote code and performed the experiments. MS contributed significantly to data analysis and manuscript preparation. DZ performed the data analyses and wrote the manuscript. QL was responsible for the overall experimental design. ZZ and JW checked the manuscript and made valuable suggestions on the data processing process. All authors contributed to the article and approved the submitted version.

## Conflict of Interest

The authors declare that the research was conducted in the absence of any commercial or financial relationships that could be construed as a potential conflict of interest.

## Publisher’s Note

All claims expressed in this article are solely those of the authors and do not necessarily represent those of their affiliated organizations, or those of the publisher, the editors and the reviewers. Any product that may be evaluated in this article, or claim that may be made by its manufacturer, is not guaranteed or endorsed by the publisher.
